# 

**Published:** 1917-12-15

**Authors:** 


Rates of Subscription: Twelve months, 6/6; Six months, 4/-; three months, 2/-, post free to any address in the United Kingdom. Abroad, Twelve months, 8/8; Six months, 5/-; Three months, 2/6, post free. THE HOSPITAL "Index" to Volume LXII., April 1917 to September 1917, is now ready, price 2½d. per copy, post free. Address The Manager, The Hospital, "The Hospital" Building, 28/29 Southampton Street, Strand, London, W.C. 2.

### Deferred for Twelve Months.

Camberwell Board of Guardians have considered an application for an increase of salary from Dr. E. W. G. Masterman, medical superintendent of their infirmary, owing to the increased cost of living. Dr. Masterman was appointed to the position for the period of the war in August 1916, his salary being £750, with unfurnished house, gas, water, and washing, the emoluments being valued at £190 a year. The Infirmary Committee, who considered his request, recommended the board to grant him an extra £80 a year, but the Guardians, by a majority of twelve to nine, decided to defer the matter for twelve months. Alderman St. Cedd maintained that after the war was over the country would be flooded with doctors ready to take up the position of superintendent at infirmaries at a much lower salary. As it is generally maintained that there will be in all branches of trade a tendency to keep wages at their present high level, surely the same should apply to those in charge of large institutions.

### The Activities of the American Red Cross.

A statement issued by the American Red Cross War Council giving the collections and disbursements up to November 1 shows that nearly £16,000,000 was collected and approximately £8,200,000 disbursed, no less than £5,400,000 being spent on the needs of the Allies. Of this, France has received £3,900,000; Belgium, £144,000; Russia, £285,000; and Rumania, £300,000; whilst smaller amounts have been given to Italy, Serbia, Armenia, Syria, and Great Britain.

